# Psychedelic Mushrooms in the USA: Knowledge, Patterns of Use, and Association With Health Outcomes

**DOI:** 10.3389/fpsyt.2021.780696

**Published:** 2022-01-03

**Authors:** Richard Matzopoulos, Robert Morlock, Amy Morlock, Bernard Lerer, Leonard Lerer

**Affiliations:** ^1^Burden of Disease Research Unit, South African Medical Research Council, Cape Town, South Africa; ^2^School of Public Health and Family Medicine, Faculty of Health Sciences, University of Cape Town, Cape Town, South Africa; ^3^YourCareChoice, Ann Arbor, MI, United States; ^4^Acumen Health Research Institute, Ann Arbor, MI, United States; ^5^Biological Psychiatry Laboratory and Hadassah BrainLabs, Hadassah – Hebrew University Medical Center, Jerusalem, Israel; ^6^Back of the Yards Algae Sciences - Parow Entheobiosciences, Chicago, IL, United States

**Keywords:** psilocybin mushroom, depression, anxiety, health insurance, healthcare resource utilization, population based survey, psychedelic

## Abstract

**Introduction:** Popular media coverage of psychedelics use, growing research into this class of compounds for psychiatry and decriminalization initiatives, are transforming the public perception of psychedelics. However, little is known about levels of knowledge and psychedelic mushroom (PM) use among American adults.

**Methods:** We examined PM use and various measures of health status, quality of life, and self-reported mental health outcome measures obtained through a national on-line, cross-sectional survey of adults with a demographic composition representative of the US adult population by region, gender, age, and race (weighted *N* = 251,297,495) from November 2020–March 2021.

**Results:** General mental health and well-being were popular reasons for PM use (63.6%), although use for medically-diagnosed (31.8%) and self-diagnosed (19.0%) conditions was also common. PM users reported more depression and anxiety as reflected in higher GAD-7 and PHQ-9 scores. Factors predictive of PM use included being male [OR 1.54 95%CI 1.09–2.15] and having higher Charlson Comorbidity Index scores [OR 1.42; 95%CI 1.22–1.65]. Self-reported PM use was less likely among participants with health insurance [OR = 0.50 (0.35–0.72)], increased age [OR = 0.92 (0.90–0.93)] and, relative to those living in the west US census region, living in the northeast [OR = 0.27 (0.15–0.50)], midwest [OR = 0.34 (0.20–0.56)], and south [OR = 0.38 (0.26–0.55)].

**Discussion and Conclusions:** A significant number of Americans are already “self-medicating” with PM and as growing positive media coverage of psychedelics drives public interest in the health benefits of PM, this number will increase. The association between PM use and poor mental health requires further research to inform policy.

## Introduction

Psychedelics (serotonergic hallucinogens) have been used for thousands of years around the world. Their psychoactive effects on perception, mood and cognition have been applied in religious ceremonies and personal healing (1). In the 1960s and 70s, pharmaceutical companies and academic institutions studied the therapeutic potential of psychedelics including psilocybin and lysergic acid diethylamide (LSD). However, by the early 70's a wave of strict prohibition swept through industrialized countries leading to the near cessation of biomedical psychedelics research for a period of almost 50 years. In the last 5 years, there has been renewed interest in the use of psychedelics and a significant acceleration in research activity, leveraging their neuroplastic effect as a novel approach for the treatment of psychiatric disorders. This was coupled with a greater openness of the US Food and Drug Administration (FDA) to research on these substances. Methylenedioxymethamphetamine (MDMA) and synthetic psilocybin-assisted psychotherapy received breakthrough therapy designation in 2017 and 2018, respectively. Esketamine, the S-enantiomer of ketamine, was approved by the FDA for treatment-resistant depression in 2019 (2). As of September 2021, 74 clinical studies are registered on clinicaltrials.gov; the majority studying psilocybin for the treatment of a variety of mental health conditions including depression, post-traumatic stress disorder (PTSD) addiction (3–5), pain and neurodegenerative disorders (6–9).

A number of cities and states in the US have effectively decriminalized psychedelics and in addition to the scientific and clinical attention, psychedelics are increasingly represented in public media and there is some concern that this wave of psychedelic decriminalization is not being accompanied by evidence-based regulation (10).

As compared to opioids, alcohol and tobacco, psychedelics have low addictive potential and benign toxicity profiles (1, 7). There are anecdotal and empirical reports of users experiencing reduced psychological distress and suicidality compared to users of other “recreational or illicit” drugs (11). Psychedelics are included within the broad category “hallucinogens” in the National Survey on Drug Use and Health (NSDUH), which collects lifetime, past-year and past month drug use estimates representative of persons aged 12 and over in each state and the District of Columbia. The most recent survey data indicate a significant increase in hallucinogen use from 4.69 million to 6.01 million between 2015 and 2019. The increase was specific to LSD (60% increase) and a 95% increase in other unspecified hallucinogens, a category that included the entheogens (psychoactive substances historically used for religious or spiritual purposes) such as mescaline and psilocybin, whereas phencyclidine (PCP) and ecstasy use declined over this period (12).

Psilocybin, which is the main psychoactive ingredient in more than 200 species of psychedelic mushrooms (PM) (13), has a particularly benign safety profile and possible positive health effects (14). Alongside the recent increase in information on its promising therapeutic use, the COVID-19 pandemic has considerably increased societal stress, which adds to the propensity to self-medicate as a coping mechanism. This has manifested in the US through an increase in drinking and alcohol related harms (15), increased anxiety, depression, and social isolation among people with substance use disorders (16), and rising addiction rates (17).

The purpose of this research was to ascertain knowledge of PM use and associations with health status and healthcare utilization among American adults. We compared socio-demographic data for PM users and non-psychedelics users and explored the associations between PM use and various measures of health status, health related quality of life, and self-reported mental health outcome measures. We also assessed whether two important contemporary events in the US that may have impacted on mental health, namely the COVID-19 pandemic and the national election and its aftermath, were associated with increased use.

## Methods

### Data Collection

Data were collected through an on-line cross sectional survey of adults (18 years or older) residing in the US in accordance with Acumen Health Research Institute's (AHRI) established survey procedure. A random stratified sampling framework ensured a community-based sample with a demographic composition representative of the US adult population by region, gender, age, and race, according to the US Census and its standard classifications (US Census American Community Survey 5-year estimate, 2015–2019). Participants were recruited through AHRI's online research panels that were fielded monthly between November 2020 and March 2021, with each fielding lasting up to 7 days. The survey targeted ~1,000–2,000 respondents per month. We applied analysis weights to account for selection probabilities. Multiple quality control processes integrated throughout data collection, included digital finger printing technologies to validate unique respondents and to ensure study data was comprised of non-fraudulent responses. A detailed methodology has been published previously (18).

### Psychedelic Use Survey Questions

All survey participants were asked three questions with regard to psychedelics, which in our survey included the following categories: PM, ayahuasca, DMT, 5-MEODMT, ibogaine, kambo, ketamine, LSD, MDMA and peyote. Participants were first asked about the context in which they have heard about psychedelics: “*Have you heard of psychedelic use for any of the following? Select all that apply*.” Response options included: general mental health and well-being when feeling basically satisfied with life or for personal development; managing a diagnosed psychiatric condition (depression, PTSD addition, etc.); to address a specific worry/concern in your life (e.g., relationship issue, bereavement, addiction, trauma); no knowledge, and other (please specify). Participants were then asked to respond to the statement, “*In the past 6 months I have heard more than usual about the positive uses of psychedelic drugs (e.g., magic mushrooms) for mental health issues (depression, PTSD, addition, etc.)*.” Five response options ranged from “strongly agree,” “agree,” “neither agree nor disagree,” “disagree,” and “strongly disagree.”

The next survey question asked about psychedelic use in the past 12 months: “*In the last 12 months have you used any of the following? Select all that apply*.” Participants could select “None of these,” or select one or more of 12 psychedelics listed. For each psychedelic selected, participants answered four “follow-on” questions about their experience with that specific psychedelic in the last 12 months. The first follow-on question was, “*In the last 12 months, did you use* <*the psychedelic*> *with the specific intention of improving your: Select all that apply*.” with response options being those in the first question (i.e., general mental health and well-being…). The next two questions asked, “*In the last 12 months, did your use of* <*the psychedelic*>*:*” increase, decrease, was not impacted by or “other (please specify)” on account of COVID-19 and election politics. The fourth question asked whether the participant sought emergency medical treatment following the use of the psychedelic and offered yes or no response options.

### Variables

Participants reported on their individual demographic characteristics, educational attainment, health status, and knowledge and use of psychedelics in the last 12 months. Estimated participant median household income was derived from US Census American Community Survey 5-year estimate 2015–2019 data for the participant's zip code. Self-reported co-morbidities were summarized according to the Charlson Comorbidity Index (CCI), a survival stratification tool that assesses the comorbidity risk associated with several conditions and estimates 10-year survival in patients suffering from multiple comorbidities (19, 20). Health-related quality of life (HRQoL) was assessed using the Veterans RAND 12-Item (VR-12) physical component summary (PCS), mental component summary (MCS), and health utility (VR-6D) (21). Anxiety was assessed with the Generalized Anxiety Disorder 7-item (GAD7) (22). Depression was assessed with the Patient Health Questionnaire 9-item (PHQ9) (23). A question about the positive uses of psilocybin “In the last 6 months I heard more than usual about the positive uses of psychedelic drugs (e.g., psychedelic mushrooms) for mental health issues (depression, PTSD, addiction, etc.)?” was rated on a 5-point Likert scale from 0 (strongly disagree) to 4 (strongly agree).

### Analysis

Weighting was carried out according to the census data based on age, sex, and region using a Taylor Series Linearization (TSL) method for estimating population characteristics controlling for complex sample survey data where the standard errors are based on the actual *N* and not the weighted *N*. Study variables were subjected to chi-square and one-way analysis of variance. PM users were compared with participants that had not taken a psychedelic in the past 12 months according to population level characteristics, views on psychedelics, treatments, co-morbidities, and HRQoL. Users of other psychedelics and combined use of PMs and another psychedelic were excluded from the comparative analysis. An unweighted multivariate logistic regression model controlling for sex, age, race, region, education, employment, CCI score, GAD7, PHQ9, and health insurance predicted psilocybin use. Odds ratios (OR) with 95% confidence intervals are reported.

### Ethics

Respondents were required to be 18 years old or older to participate in the study. Participants confirmed their voluntary agreement to participate and were informed they could leave the survey at any time. Each participant was compensated for their time spent participating according to a reward system based on marketplace points. Participants who fully completed our survey received points worth between $1.40 and $2.45. The actual amount of points awarded to each respondent was determined by the research panel's incentive policy.

The survey was deemed Institutional Review Board-exempt as all responses were anonymized, aggregated, and could not be related back to individual participants.

## Results

A total of 7,139 participants were included in the sample—a response rate of 83.8%. Psychedelic use in the last year was confirmed by 526 participants (or 7.4% of the total unweighted sample), which included 134 participants (1.9%) who had used PMs and other psychedelics and 122 (1.7%) who had used PMs exclusively. Weighted to the US adult population this represented 7.1% or 17,862,102 adult psychedelic users overall (95%CI: 6.5–7.7), of which 49.2% or 8,780,328 adults (95%CI: 44.7–53.6) were PM users.

We compared the 4,121,102 (1.7%, 95%CI: 1.4–2.1) adults who used only PMs with 233,435,392 (98.3%, 95%CI: 97.9–98.6) adults who did not use any psychedelics (Table 1). In descriptive unadjusted comparisons PM users were significantly more likely to be male (68.3% [95%CI: 59.3–76.1] compared to 47.2% [95%CI: 45.9–48.5] among non-psychedelic users), younger (mean age 38.1 [SD 10.8] vs. 48.6 [SD 16.6] years), Hispanic/Latino/Spanish (16.5 [95%CI: 10.6–24.7] vs. 8.1% [95%CI: 7.4–8.9]) and reside in the US's Western Region (33.8 [95%CI: 25.5–43.2] vs. 23.0% [95%CI: 21.8–24.1]). Other demographic and socio-economic measures such as income, employment and educational attainment were similar.

**Table 1:** Descriptive non-psychedelic user and PM user characteristics, US Adult population 2020/21 (weighted).

	**Sig**.	**Non-psychedelic user**	**PM user**
Sample, % (*n*)		98.3% (233,435,392)	1.7% (4,121,102)
**Demographics**
Female, % (95%CI)	^***^	52.8 (51.5–54.1)	31.7 (23.9–40.7)
Male, % (95%CI)		47.2 (45.9–48.5)	68.3 (59.3–76.1)
Age in years, mean (SD)	^***^	48.56 (16.60)	38.12 (10.83)
Black or African American, % (95%CI)		11.5 (10.8–12.2)	9.6 (5.8–15.6)
White, % (95%CI)		73.6 (72.4–74.8)	80.4 (72.0–86.8)
Other, % (95%CI)^+^		14.9 (13.8–16.0)	10.0 (5.3–18.0)
Hispanic/Latino/Spanish origin, % (95%CI)	^**^	8.1 (7.4–8.9)	16.5 (10.6–24.7)
Northeast, % (95%CI)	^*^	17.8 (16.8–18.8)	11.1 (6.4–18.7)
Midwest, % (95%CI)		21.1 (20.1–22.1)	17.2 (11.2–25.4)
South, % (95%CI)		38.1 (36.9–39.4)	37.9 (29.5–47.1)
West, % (95%CI)		23.0 (21.8–24.1)	33.8 (25.5–43.2)
**Socioeconomics**
Education beyond high school, % (95%CI)		73.4 (72.3–74.5)	80.4 (72.2–86.6)
Employed ≥32 hours/week, % (95%CI)		30.2 (29.0–31.4)	34.4 (26.2–43.7)
Household income by area-level median, mean (SD)		65,525 (26,302)	62,929 (25,452)

* *p < 0.05*;

** *p < 0.01; and*

*** *p < 0.001*.

+ *Includes American Indian or Alaska Native, Asian, Native Hawaiian or Other Pacific Islander according to US Census classifications available at https://www.census.gov/topics/population/race/about.html*.

In terms of health status (Table 2), descriptive unadjusted comparisons show PM users significantly less likely to be overweight than non-users, but reported significantly higher levels of certain comorbid conditions, namely depression, anxiety, migraines, and insomnia. Overall quality of life (VR-12) was also lower for PM users as measured by lower mental health scores (39.5 [SD 11.3] vs. 45.5 [SD 12.4]) and health utility (0.63 [SD 0.10] vs. 0.69 [SD 0.12]), and PM users reported higher levels of anxiety (GAD-7 scores of 9.6 [SD 5.8] vs. 5.9 [SD 5.8]) and depression (PHQ-9 scores of 11.2 [SD 7.0] vs. 6.8 [SD 6.8]) than non-users.

**Table 2:** Descriptive non-psychedelic user and PM user health, quality of life and health-seeking behavior, US Adult population 2020/21 (weighted).

	**Sig**.	**Non-psychedelic user**	**PM user**
**Health**			
Body mass index (BMI) in lbs/in^2^, mean (SD)	^***^	28.16 (7.11)	26.01 (5.43)
Comorbidities^+^, mean (SD)		4.07 (3.66)	4.20 (3.10)
**Specific comorbidities^++^**			
Depression, % (95%CI)	^***^	28.3 (27.2–29.5)	56.8 (47.5–65.7)
Anxiety, % (95%CI)	^***^	32.8 (31.6–34.0)	49.1 (40.0–58.3)
Chronic pain incl. back, neck, % (95%CI)		44.5 (43.3–45.8)	46.7 (37.6–55.9)
Allergies, % (95%CI)		28.7 (27.6–29.9)	32.2 (24.3–41.1)
Migraines or severe headaches, % (95%CI)	^***^	18.6 (17.6–19.6)	31.2 (23.3–40.5)
Insomnia, % (95%CI)	^*^	20.6 (19.6–21.7)	30.0 (22.2–39.0)
Hypertension, % (95%CI)		23.6 (22.5–24.7)	16.8 (11.0–24.7)
Diarrhea, chronic, or more than occasional, % (95%CI)		14.2 (13.3–15.1)	10.7 (6.3–17.5)
Sleep apnea, % (95%CI)		8.2 (7.5–8.9)	10.1 (5.8–17.1)
Gastroesophageal reflux disease (GERD), % (95%CI)		13.6 (12.7–14.5)	9.7 (5.3–16.9)
Constipation, chronic, or more than occasional, % (95%CI)		13.7 (12.8–14.6)	8.9 (5.0–15.3)
Hyperlipidemia, % (95%CI)		15.5 (14.6–16.5)	8.7 (4.6–15.6)
CCI score, mean (SD)		0.48 (1.04)	0.60 (0.98)
Anxiety (GAD-7 score), mean (SD)	^***^	5.88 (5.79)	9.62 (5.76)
Anxiety (GAD-7 ≥10), % (95%CI)	^***^	25.3 (24.2–26.4)	50.2 (41.0–59.3)
Depression (PHQ-9 score), mean (SD)	^***^	6.75 (6.79)	11.23 (6.99)
Depression (PHQ-9 ≥10), % (95%CI)	^***^	29.5 (28.4–30.7)	56.1 (46.6–65.0)
**Health-related quality of life**			
**Veterans-RAND 12-item (VR-12)**			
Mental health composite score (MCS), mean (SD) (t-score)	^***^	45.53 (12.44)	39.49 (11.25)
Physical health composite score (PCS), mean (SD) (t-score)	^*^	45.28 (10.45)	43.51 (9.24)
Health utility (VR-6D), mean (SD)	^***^	0.69 (0.12)	0.63 (0.10)
**Health-seeking behavior**			
Primary care physician visit, % (95%CI)		52.4 (51.1–53.7)	51.1 (41.9–60.3)
Specialist, % (95%CI)		28.6 (27.5–29.8)	23.2 (16.4–31.7)
Other healthcare provider, % (95%CI)^+++^	^*^	19.7 (18.7–20.7)	28.2 (20.7–37.1)
Urgent care, % (95%CI)^++++^	^***^	9.9 (9.2–10.7)	20.8 (14.3–29.3)
Outpatient procedure or surgery, % (95%CI)		8.6 (7.9–9.3)	7.2 (3.5–14.2)
Emergency department, % (95%CI)^+++++^		10.1 (9.4–10.9)	12.9 (8.1–20.1)
Hospitalization, % (95%CI)	^**^	3.9 (3.4–4.4)	9.2 (5.2–15.8)

* *p < 0.05*;

** *p < 0.01; and*

*** *p < 0.001*.

+ *Comorbid conditions were self-reported and experienced within the past year*.

++ *The 10 most reported conditions for each cohort are reported in descending order for the PM cohort*.

+++ *Other healthcare providers include nurses, pharmacists, and other allied health professionals*.

++++ *Urgent care is care for non-life-threatening emergencies (e.g., an injury that does not appear life threatening but cannot wait until the next day) provided in an urgent care setting*.

+++++ *Emergency departments provide care for life-threatening emergencies*.

PM users consistently and significantly held more favorable views of the positive potential for PM use for the treatment of a range of conditions than non-users (Table 3), including for general mental health, well-being and personal development (65.8 [95%CI: 56.7–73.9] vs. 15.8% [95%CI: 14.9–16.7]), and managing diagnosed (50.9 [95%CI: 41.7–60.1] vs. 16.6% [95%CI: 15.7–17.6]) and self-diagnosed psychiatric conditions (34.7 [95%CI: 26.5–44.0] vs. 7.6% [95%CI: 7.0–8.3]). This was consistent with their rationale for using PMs, with general mental health and well-being being the most common (63.6% [95%CI: 54.2–72.0]), followed by use for diagnosed psychiatric conditions (31.8% [95%CI: 23.9–41.0]) and self-diagnosed conditions (19.0% [95%CI: 13.0–27.0]). PM users were also significantly more likely than non-users to have heard more frequent positive reporting of the use of psychedelic drugs for mental health issues (depression, PTSD, addiction, etc.) in the last 6 months than previously (mean score 2.06 [SD 1.07] vs. 3.56 [SD 1.20] out of a scale of 1 “strongly agree” to 5 “strongly disagree”). Frequency of use did not seem to have been influenced on account of either COVID-19 or election politics. Medical treatment following use reported by 17.9% [95%CI: 11.9–26.0] of PM users.

**Table 3:** Non-psychedelic user and PM user, knowledge and use of PMs for health and well-being, US Adult population 2020/21 (weighted).

	**Sig**.	**Non-psychedelic user**	**PM user**
**Health and well-being knowledge**			
More frequent positive information about PM use for mental health in last 6 months (score) (range: 1, strongly agree to 5, strongly disagree), mean (SD)	^***^	3.56 (1.20)	2.06 (1.07)
Positive potential for general mental health, well-being and personal development (preventive), % (95%CI)		15.8 (14.9–16.7)	65.8 (56.7–73.9)
Positive potential managing a diagnosed psychiatric condition—PTSD, depression, addiction, etc. (curative), % (95%CI)		16.6 (15.7–17.6)	50.9 (41.7–60.1)
Positive potential managing a self-diagnosed condition/concern—relationship issue, bereavement, trauma, addiction, etc. (curative), % (95%CI)	^***^	7.6 (7.0–8.3)	34.7 (26.5–44.0)
No knowledge, % (95%CI)	^***^	69.7 (68.5–70.9)	13.6 (8.3–21.4)
**Reasons for use**			
General mental health, well-being, and personal development (preventive), % (95%CI)	–	–	63.6 (54.2–72.0)
Managing a diagnosed psychiatric condition—PTSD, depression, addiction, etc. (curative), % (95%CI)	–	–	31.8 (23.9–41.0)
Managing a self-diagnosed condition/concern—relationship issue, bereavement, trauma, addiction, etc. (curative), % (95%CI) ^+^	–	–	19.0 (13.0–27.0)
**Frequency of use**			
Increased on account of COVID-19	–	–	14.7 (9.4–22.3)
Decreased on account of COVID-19	–	–	17.9 (11.9–25.9)
No impact	–	–	66.6 (57.5–74.7)
Increased on account of election politics	–	–	13.1 (8.1–20.5)
Decreased on account of election politics	–	–	11.6 (7.0–18.7)
No impact	–	–	74.3 (65.5–81.5)
**Potential harm**			
Seeking medical treatment following use	–	–	17.9 (11.9–26.0)

*p < 0.05; **p < 0.01; and

*** *p < 0.001*.

+ *Most common responses included recreation, mental relaxation, and social anxiety*.

**Figure 1:**
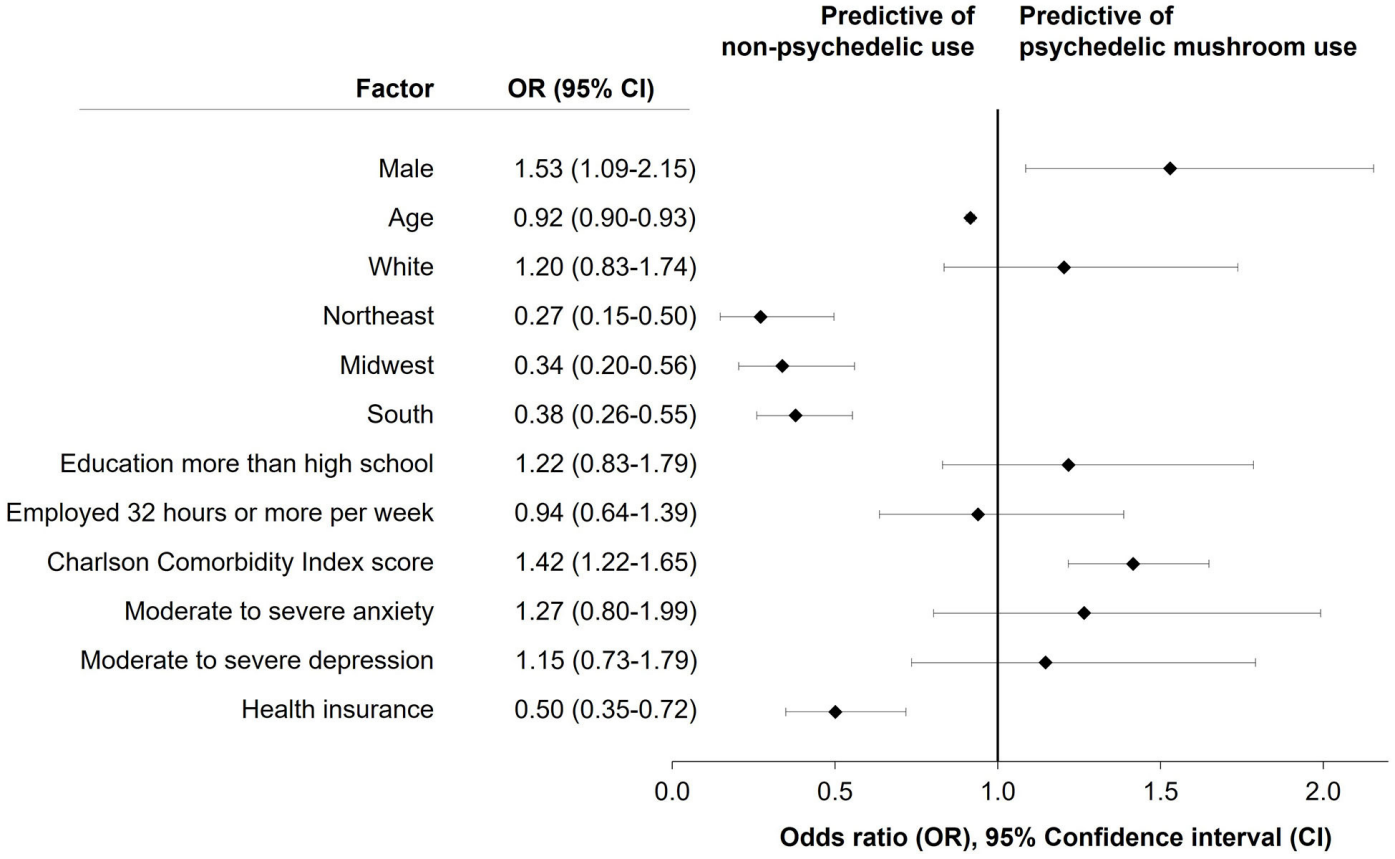
Results of multivariate logistic regression model predicting past year psychedelic mushroom use for selected demographic and educational factors and health and comorbidity indicators.

The multivariate logistic regression analysis explored the correlation between various factors and PM use (Figure 1). Factors predictive of PM use included being male [OR = 1.53 (1.09–2.15)] and having a higher CCI (more comorbid conditions) [OR = 1.42 (1.22–1.65)]. Those with health insurance [OR = 0.50 (0.35–0.72)], increased age [OR = 0.92 (0.90–0.93)], and relative to those living in the west US census region, those living in the northeast [OR = 0.27 (0.15–0.50)], midwest [OR = 0.34 (0.20–0.56)], and south [OR = 0.38 (0.26–0.55)] were less likely to report PM use.

## Discussion

The objective of this study was to assess knowledge about PMs among American adults and explore associations between PM use and various general and self-reported mental health outcome measures. Our findings confirm the popularity of psychedelics more broadly and PMs specifically among the US adult population (13, 24, 25). Estimated past-year psychedelic use of 7.7%, which equated to ~17.9 million adult Americans (95%CI: 16.4–19.4 million) was almost three times higher than recorded in the NSDUH, which estimated past-year hallucinogen use of just more than 6 million persons in 2019 (12).

The popularity of PMs amongst psychedelics users, approximately half of all past year psychedelic use in our study, was not unexpected. PMs have a relatively benign reputation among psychedelics generally and there is growing media attention to the health and wellness benefits of mushrooms. Among the different classes of psychedelics, classic tryptamines, which include psilocybin, are uniquely associated with a decreased likelihood of psychological distress and suicidal thinking and are widely considered to hold the greatest therapeutic potential among lifetime users (26). This was confirmed by PM users identified in our study. Despite this favorable view PM users reported more anxiety, depression, and comorbidities and higher utilization of healthcare resources. Although associations for anxiety and depression were not significantly associated with PM use in the logistic regression analysis, PM users had significantly higher CCI scores and were less likely to have health insurance.

These findings were distinctly different from those of a recent international study which showed that regular users of psychedelic drugs had less psychological stress compared to occasional users and non-users, suggesting that the use of psychedelics might either be a protective factor or that people with certain traits were more prone to frequently use psychedelic drugs (27). This difference could be ascribed to our study focusing on any use rather than regular use, or that PM users in the US comprise a distinctly different demographic group as compared to their international counterparts. Given the lower rates of health insurance, PM users may be more likely to self-medicate.

Classical serotonergic psychedelics do not cause neurotoxic effects, organ damage or long-term neuropsychological deficits (1). Acute psychological effects can be controlled through supervised administration of perception-altering doses. The risk/benefit of sub-perceptual doses or molecules lacking perceptual effects remains to be formally studied (28). There seems to be limited possibility of physical dependence. Animal studies have shown that they lack reinforcing properties that induce self-administration, which limits the potential for abuse as well as withdrawal symptoms (29, 30). This is consistent with a US population-based study that indicated no association between lifetime use of psychedelics generally, or specific psychedelics including psilocybin, and addiction, increased rate of mental illness and prolonged, or re-occurring, perceptual effects (31).

Less information is available for novel psychedelics, which are becoming increasingly popular and seemingly less benign. Sexton et al. found a greater likelihood of past year suicidal thinking and past year suicidal planning among lifetime users of novel psychedelics (32). Subsequent analysis of the NSDUH found that novel phenethylamine use specifically was associated with increased suicidal thinking and suicidal planning (26).

Across cultures, people try to make sense of the world around them, including how likely it is that a negative outcome will occur for them and what will increase or reduce that risk—*lay epidemiology* (33). Their decisions are complicated not only by the expanding variety of available psychedelics, but also their potential application for an increasing range of therapeutic uses. It is known, for example, that psychedelics exert significant modulatory effects on immune responses by altering signaling pathways involved in inflammation, cellular proliferation, and cell survival (34). Preclinical research has shown that psychedelics promote structural and functional neural plasticity in key brain regions linked to psychological functioning (9). A recent publication provides the scientific hypothesis for low doses of psychedelics exerting effects on mental well-being and neurological healing through indirect modulation of the gut-brain axis (35). Therapies involving psychedelics are among the innovative, potential treatments for traumatic brain injury (36).

The use of an online data collection platform reduced the risk of stigma by ensuring privacy and confidentiality, which may explain the considerably higher past year estimates than were reported in other surveys. Although we made every effort to avoid sampling biases and to ensure the validity of information, standard survey limitations arising from self-reporting still applied and may have been amplified by including only respondents who had access to a computer and were willing to take an online survey. Another limitation was that comparisons were made with no alpha adjustment resulting in an increased risk of Type 1 errors. We excluded users of other psychedelics and users that combined PM use with use of other psychedelics to avoid confounding, but in so doing may have missed other significant associations. The inability to distinguish between different users may have introduced some selection bias—people using psychedelics parsimoniously for the first time were more likely to be included, whereas experienced “psychonauts” using a wide range of designer psychedelics could have been excluded (37). We also note that the timing of the study was unusual, in that it was conducted at the height of the COVID-19 pandemic, which may make the findings less generalizable to surrounding time periods. COVID-19 has thrown mental health disorders into sharp focus and therapeutic interventions are likely to find renewed impetus. Almost one-third of COVID-19 cases with serious complications may experience PTSD (38) while the full impact on grieving families and communities will only become apparent in the future.

## Conclusions

The present research clearly demonstrates the utility of a population-based understanding of psychedelics use, among which self-medication with PMs remains particularly popular for wellness and mental health. Despite the overwhelmingly positive reputation of lifetime use of PMs in our study, the association with beneficial health outcomes remains unclear. The extent to which uptake is influenced by emerging scientific evidence versus anecdotal or pseudoscientific knowledge is incidental to the need for the development of guidelines to optimize therapeutic use. Future research should include regularized application of population-level methodology surveys to assess changes over time as well as additional modules to identify key drivers of use and misuse for different psychedelics, and to indicate possible therapeutic applications for specific conditions.

## Data Availability Statement

The raw data supporting the conclusions of this article are available online. 10.5061/dryad.bzkh189b6.

## Ethics Statement

Ethical review and approval was not required for the study on human participants in accordance with the local legislation and institutional requirements. The patients/participants provided their written informed consent to participate in this study.

## Author Contributions

RMa drafted the manuscript and summarized the findings. RMo and AM informed methodology, conducted analyses, and were responsible for ensuring analyses were interpreted correctly. LL conceptualized the study and along with BL, contributed meaningful pharmacological expertise to aid in the interpretation of results. All authors contributed to and approved of the final version of the manuscript.

## Funding

BYAS sponsored—Back of the Yards Algae Sciences, Chicago-based sustainable biotechnology company.

## Conflict of Interest

RMo was employed by company YourCareChoice. BL and LL hold equity in Back of the Yards Algae Sciences and Parow Entheobiosciences. The remaining authors declare that the research was conducted in the absence of any commercial or financial relationships that could be construed as a potential conflict of interest.

## Publisher's Note

All claims expressed in this article are solely those of the authors and do not necessarily represent those of their affiliated organizations, or those of the publisher, the editors and the reviewers. Any product that may be evaluated in this article, or claim that may be made by its manufacturer, is not guaranteed or endorsed by the publisher.
