# Corrigendum: Psychedelic Mushrooms in the USA: Knowledge, Patterns of Use, and Association With Health Outcomes

**DOI:** 10.3389/fpsyt.2022.877390

**Published:** 2022-03-23

**Authors:** Richard Matzopoulos, Robert Morlock, Amy Morlock, Bernard Lerer, Leonard Lerer

**Affiliations:** ^1^Burden of Disease Research Unit, South African Medical Research Council, Cape Town, South Africa; ^2^School of Public Health and Family Medicine, Faculty of Health Sciences, University of Cape Town, Cape Town, South Africa; ^3^YourCareChoice, Ann Arbor, MI, United States; ^4^Acumen Health Research Institute, Ann Arbor, MI, United States; ^5^Biological Psychiatry Laboratory and Hadassah BrainLabs, Hadassah – Hebrew University Medical Center, Jerusalem, Israel; ^6^Back of the Yards Algae Sciences - Parow Entheobiosciences, Chicago, IL, United States

**Keywords:** psilocybin mushroom, depression, anxiety, health insurance, healthcare resource utilization, population based survey, psychedelic

In the original article, there was an error. In our discussion we noted “past year use of all hallucinogens other than LSD, PCP and ecstasy at <1 million persons” in the 2019 NSDUH. We had not considered that the specific NSDUH survey question permitted multiple responses that were not mutually exclusive. As PM use was not specifically listed it could therefore have accounted for any number of the 5.2 hallucinogen users. We acknowledge that the statement is therefore an error of fact. However, given that past-year psychedelic use for all psychedelics recorded in our survey was almost three times higher than recorded in the NSDUH, we consider it extremely unlikely that reporting of PM usage would be so differential as to materially change the observation that our online data collection platform provided considerably higher estimates of past year psychedelic use than reported in surveys.

A correction has been made to **Discussion**, paragraph one:

“The objective of this study was to assess knowledge about PMs among American adults and explore associations between PM use and various general and self-reported mental health outcome measures. Our findings confirm the popularity of psychedelics more broadly and PMs specifically among the US adult population (13, 24, 25). Estimated past-year psychedelic use of 7.7%, which equated to ~17.9 million adult Americans (95%CI: 16.4–19.4 million) was almost three times higher than recorded in the NSDUH, which estimated past-year hallucinogen use of just more than 6 million persons in 2019 (12).”

A correction has also been made to **Methods**, **Psychedelic Use Survey Questions**, paragraph one:

“All survey participants were asked three questions with regard to psychedelics, which in our survey included the following categories: PM, ayahuasca, DMT, 5-MEO DMT, ibogaine, kambo, ketamine, LSD, MDMA and peyote. Participants were first asked about the context in which they have heard about psychedelics: “*Have you heard of psychedelic use for any of the following? Select all that apply*.” Response options included: general mental health and well-being when feeling basically satisfied with life or for personal development; managing a diagnosed psychiatric condition (depression, PTSD addition, etc.); to address a specific worry/concern in your life (e.g., relationship issue, bereavement, addiction, trauma); no knowledge, and other (please specify). Participants were then asked to respond to the statement, “*In the past 6 months I have heard more than usual about the positive uses of psychedelic drugs (e.g., magic mushrooms) for mental health issues (depression, PTSD, addition, etc.)*.” Five response options ranged from “strongly agree,” “agree,” “neither agree nor disagree,” “disagree,” and “strongly disagree.””

The authors apologize for this error and state that this does not change the scientific conclusions of the article in any way. The original article has been updated.

## Publisher's Note

All claims expressed in this article are solely those of the authors and do not necessarily represent those of their affiliated organizations, or those of the publisher, the editors and the reviewers. Any product that may be evaluated in this article, or claim that may be made by its manufacturer, is not guaranteed or endorsed by the publisher.

